# Genomic Subtypes and Computational Biomarkers in Non-Muscle-Invasive Bladder Cancer Guiding Optimal Timing of Radical Cystectomy and BCG Response Prediction

**DOI:** 10.3390/genes17020153

**Published:** 2026-01-29

**Authors:** Vlad-Horia Schițcu, Vlad Cristian Munteanu, Mihnea Bogdan Borz, Ion Cojocaru, Octavia Morari, Mircea Gîrbovan, Andrei-Ionuț Tișe

**Affiliations:** 1 Department of Urology, Institute of Oncology “Prof. Dr. Ion Chiricuta” Cluj-Napoca, 400015 Cluj-Napoca, Romania; schitcu@yahoo.com (V.-H.S.); vladcristian.munteanu@gmail.com (V.C.M.); mo.octavia@yahoo.com (O.M.); 2 Department of Urology, Targu Mures County Emergency Clinical Hospital, 540136 Targu Mures, Romania; mircea_girbovan_98@yahoo.com; 3 Department of Urology, Galati County Emergency Hospital, 800578 Galati, Romania; cojocaruion90@yahoo.com; 4 Department of Urology, Alba County Emergency Hospital, 711325 Alba-Iulia, Romania; andreitisemd@gmail.com

**Keywords:** non-muscle-invasive bladder cancer, radical cystectomy, BCG response prediction, molecular biomarkers, genomic subtypes, timing of surgery, precision oncology

## Abstract

Non-muscle-invasive bladder cancer (NMIBC) accounts for approximately 70% of newly diagnosed bladder cancer cases but exhibits significant clinical heterogeneity in treatment response and progression risk. While intravesical bacillus Calmette–GuérinCa (BCG) therapy remains the gold standard for high-risk disease, approximately 30–50% of patients experience BCG failure, creating a critical decision point between additional bladder-sparing therapy (BST) and early radical cystectomy (RC). Recent clinical data from the CISTO study suggest that, in appropriately selected patients, RC may be associated with higher 12-month recurrence-free survival while maintaining comparable cancer-specific survival and physical functioning. In this narrative review, we synthesize contemporary evidence on NMIBC genomic and transcriptomic subtypes, immune contexture, and clinicopathologic features associated with BCG response and progression risk, with emphasis on clinically oriented classification systems such as BCG Response Subtypes (BRS1–3) and UROMOL21. We highlight how tumor-intrinsic biology (e.g., EMT-associated programs), immune phenotypes (inflamed vs. immune-cold microenvironments), and genomic alterations may help refine risk stratification beyond traditional clinicopathologic models. To facilitate clinical integration, we propose a conceptual decisional framework that combines molecular subtype assignment, immune profiling, key pathologic risk factors, and patient considerations to generate probabilistic risk tiers that support selection among early RC, BST, and clinical trial strategies. Standardized multicenter cohorts and prospective evaluation are needed to validate integrated models and define their clinical utility for the precision timing of cystectomy in BCG-unresponsive NMIBC.

## 1. Introduction

Bladder cancer represents the 10th most diagnosed malignancy worldwide, with approximately 573,000 new cases annually [1]. Approximately 75% of newly diagnosed patients present with non-muscle-invasive disease confined to the urothelium or lamina propria (Ta, T1, or carcinoma in situ [CIS]). While traditionally considered more favorable than muscle-invasive bladder cancer (MIBC), NMIBC carries unpredictable clinical trajectories: approximately 50–70% of patients experience disease recurrence, and 10–20% progress to MIBC, with profound implications for oncologic outcomes and quality of life [2].

Since the 1980s, intravesical bacillus Calmette–Guérin (BCG) has remained the therapeutic cornerstone for high-risk NMIBC following transurethral resection of the bladder tumor (TURBT). BCG therapy achieves durable disease control in 60–70% of treated patients through endogenous antitumor immunity [3]. However, BCG failure affects 30–50% of adequately treated patients, creating a clinical impasse: historically, radical cystectomy represented the only curative option, yet many patients have declined this morbid procedure, whereas others with inherently aggressive disease experienced delayed intervention with adverse consequences [4].

The fundamental limitation in current NMIBC risk stratification derives from reliance on clinical and pathological parameters established decades ago. With C-indices for progression prediction ranging from 0.53 to 0.68, traditional risk models like the Spanish Urological Club for Oncological Treatment (CUETO) and the European Organization for Research and Treatment of Cancer (EORTC) show limited discriminatory capacity and tend to overestimate short-term risks while underestimating long-term ones [5,6]. Treatment choices are directly impacted by this prognostic uncertainty: while some patients undergo needless cystectomy despite the possibility of long-term bladder-preserving disease control, others go through numerous bladder-sparing therapies before surgery and suffer negative outcomes of upstaging and disease progression.

Our understanding of NMIBC biology has been substantially advanced by recent developments in molecular profiling, which has shown that NMIBC is a diverse group of unique molecular subtypes with varying biological behaviors and therapeutic targets. Most significantly, molecular stratification systems demonstrate superior prognostic discrimination compared with traditional clinicopathological approaches and exhibit strong association with differential BCG responsiveness [7]. Furthermore, recent prospective comparative effectiveness studies have provided evidence regarding the indication of radical cystectomy in BCG-unresponsive disease, suggesting that, in selected patients, radical surgery could yield superior recurrence-free survival while maintaining comparable cancer-specific survival and functional equivalence to bladder-sparing approaches [8].

This review synthesizes current evidence on NMIBC genomic, molecular, immunologic, and pathological biomarkers with their integration in a computational framework for predicting BCG failure and optimizing cystectomy timing in BCG-unresponsive disease.

## 2. Methods

This article is a narrative (scoping) review focused on clinically actionable molecular subtyping and biomarker frameworks in non-muscle-invasive bladder cancer (NMIBC), with emphasis on predicting response to BCG and informing the timing of radical cystectomy. We searched PubMed/MEDLINE, Embase, Web of Science, and the Cochrane Library for English-language studies published from January 2010 to December 2025. Search terms combined controlled vocabulary and keywords related to NMIBC and molecular stratification (e.g., “non-muscle invasive bladder cancer”, “NMIBC”, “molecular subtype”, “transcriptomic”, “UROMOL”, “BCG response subtype”, “BRS”, “immune contexture”, “radical cystectomy timing”, “BCG-unresponsive”). We also screened references of key reviews and clinical guidelines.

Two authors screened titles/abstracts and disagreements were resolved by consensus between authors. We prioritized NMIBC cohorts with transcriptomic/genomic subtyping that were related to relevant clinical endpoints (recurrence, progression, BCG response); studies describing immune phenotypes associated with intravesical therapy outcomes; and prospective comparative effectiveness studies addressing management after BCG failure. We excluded purely preclinical studies, case reports, and studies limited to muscle-invasive disease without separable NMIBC results.

From the eligible studies, we extracted cohort characteristics, assays/platforms (e.g., RNA-seq, targeted expression panels, IHC), subtype definitions, endpoints, and reported performance metrics (e.g., AUC, C-index, hazard ratios). Findings are presented as a narrative synthesis with an emphasis on strengths, limitations, and translation barriers (assay feasibility, harmonization, and external validation).

## 3. NMIBC Molecular Subtypes and Classification Systems

### 3.1. Luminal–Basal Paradigm

The early molecular classification systems that identified two intrinsic subtypes of bladder cancer—luminal and basal—served as the initial molecular prognostic factor for bladder cancer [9]. Luminal tumors typically present as low-grade tumors and are characterized by FGFR3 mutations (40–70% of luminal NMIBC) and activating mutations in HRAS and PIK3CA while expressing uroplakin genes (UPK1A, UPK2, UPK3A) and differentiation transcription factors (GATA3, FOXA1, PPARΓ, ELF3) [10,11]. On the other hand, basal tumors express basal cell markers (KRT5, KRT6, CD44, p63), harbor TP53 and RB1 mutations, and demonstrate chromosomal instability, which make them more susceptible to present aggressive phenotypes [12].

After a transcriptional analysis of 460 NMIBC tumors, Hedegaard et al. discovered three major molecular subclasses with distinct biological characteristics that can predict different clinical outcomes [13]. Class 1 and Class 2, both shared luminal-like characteristics but were associated with diametrically opposed clinical trajectories. While Class 1 tumors were usually associated with a good prognosis, patients harboring Class 2 tumors manifested poor progression-free survival, representing 68% of tumors with high EORTC risk scores and a profound tendency for progression, accounting for 81% of all tumors that advanced to muscle-invasive disease in the cohort. From a molecular standpoint, Class 2 tumors were strongly and significantly associated with the presence of APOBEC-related mutations and the activation of key cancer driver genes such as TP53 and ERBB2, which explains their aggressive clinical course and tendency toward progression [13].

Class 3 NMIBC is believed to be a molecularly distinct dormant cell state that is characterized by basal-like features, including the expression of KRT5 and KRT14, manifesting repressed cell-cycle activity and pronounced expression of long non-coding RNAs (lncRNAs) [13].

### 3.2. UROMOL21 Stratification

Building upon the luminal–basal framework, the comprehensive UROMOL21 classification analyzed 834 unselected NMIBC patients using integrated multi-omics, identifying four molecular classes with distinct prognostic implications (Table 1) [14].

Critically, UROMOL21 transcriptomic stratification outperformed T1HG classification for progression prediction, achieving 89% sensitivity versus 69% for conventional staging [14].

### 3.3. BCG Response Subtypes (BRS1–3)

After molecular profiling of a cohort of 132 patients with BCG-naïve high-risk NMIBC and 44 patients with recurrences after BCG (34 matched), three distinct BCG response subtypes (BRS1–3) were described [7].

BRS1 tumors (~40% of high-risk NMIBC) demonstrated an 85% BCG response rate and exhibited upregulated metabolic and cell-cycle genes involved in mycobacterial processing. With an 82% response rate, BRS2 demonstrated intermediate characteristics, while BRS3 tumors (~20% of cases) showed only a 68% response rate and accounted for most post-BCG recurrences.

Pronounced epithelial-to-mesenchymal transition (EMT) signatures, marked immunosuppression characterized by elevated regulatory T cells (Tregs) and myeloid-derived suppressor cells (MDSCs), and alternatively activated macrophages are the hallmarks of BRS3 tumors that could explain its clinical phenotype [7].

Being of clinical use, a validated gene expression signature can accurately predict BRS3 tumors with an area under the receiver operating characteristic curve of 0.87, providing clinically feasible prospective patient stratification and mindful BCG prescription [7].

### 3.4. Contemporary Four-Subtype Genomic Classification

Recent work from a Chinese cohort (where exposure to aristolochic acid is elevated) identified four genomic subtypes with distinct mutational drivers and therapeutic implications, including an aristolochic acid-associated (AA-like) subtype with characteristic mutational signatures [15]. FGFR3 mutations are present in two other subtypes, with the FGFR3/HRAS subtype representing the most common NMIBC subtype with elevated recurrence risk despite lower disease stage and the FGFR3/chromosome 9 deletion (FGFR3&chr9Del) that combines these alterations with intermediate features. The fourth subtype, genome instability (GI), alongside the AA-like subtype, demonstrated superior checkpoint inhibitor responsiveness, suggesting distinct therapeutic vulnerabilities based on mutational profiles and immune contexture [15].

### 3.5. Tumor-Stage pT1 Molecular Profiling

Molecular profiling of pT1 NMIBC identified specific transcriptomic subtypes that are prone to BCG failure, most notably the T1-Myc and T1-Early groups; the latter exhibits a 38% recurrence rate within only six months of initiating therapy [16].

Additionally, the T1-LumGU subtype presents susceptibility to BCG failure that is suspected due to its high E2F1 and EZH2 expression and frequent association with concurrent CIS, whereas the T1-TLum subtype (characterized by high FGFR3) appears significantly more responsive to treatment with no recorded recurrences in this cohort [16,17].

### 3.6. Immune Contexture

Concurrent genomic and transcriptomic profiling performed on primary tumor tissue from a cohort of 785 patients, of whom 220 had NMIBC, revealed a paradoxical immune landscape in bladder cancer, where markers of an active T-cell response confer a favorable prognosis, while markers of T-cell exhaustion (PD-1/PD-L1) are, counterintuitively, the strongest predictors of recurrence and progression [18]. This is a clinically relevant dichotomy, as the study noted an opposite trend in muscle-invasive bladder cancer (MIBC), where high expressions of PD-1 and PD-L1 correlated with a lower risk of recurrence [18].

## 4. Molecular Determinants of BCG Response Heterogeneity

### 4.1. FGFR3 Mutations and Co-Mutational Context

FGFR3 mutations represent the single most common genetic alteration in NMIBC (49–84% prevalence versus 8–20% in MIBC), typically involving activating kinase domain mutations resulting in constitutive signaling through MAPK and PI3K pathways [11]. Paradoxically, despite the association with a lower grade and stage at presentation, FGFR3-mutant NMIBC demonstrates surprisingly high recurrence risk, particularly in low-grade Ta tumors, where FGFR3 mutations independently predict recurrence in multivariable analyses [11].

Ninomiya et al. demonstrated that FGFR3 protein expression was significantly downregulated in MIBC compared to NMIBC (*p* = 0.002), establishing that the downregulation of FGFR3 is a crucial molecular event driving bladder cancer progression [19].

Critically, the co-mutation context substantially modulates prognostic significance: FGFR3-mutant tumors harboring concurrent CDKN2A co-alterations demonstrated substantially worse high-grade recurrence-free survival, while FGFR3 with CDKN1A co-alterations were associated with impaired progression-free survival even with adequate BCG therapy [11].

### 4.2. Epithelial-to-Mesenchymal Transition and BCG Resistance

EMT is believed to be an important mechanism linking molecular phenotype to BCG resistance. For example, BRS3 tumors exhibit pronounced EMT signatures with an upregulation of EMT transcription factors (ZEB2, TWIST2, SNAI3) and downregulation of E-cadherin, with an associated loss of miR-200 family expression [7]. EMT activation may suppress tumor necrosis factor-related apoptosis-inducing ligand expression, impairing BCG-induced apoptosis of tumor cells. The intimate relationship between EMT status and immunosuppressive infiltration—with elevated Tregs, MDSCs, and alternatively activated macrophages characteristic of BRS3 tumors—suggests that EMT reversal combined with immune checkpoint inhibition may sensitize BCG-resistant tumors to therapy [7].

### 4.3. Immune Contexture and T-Cell Infiltration

A favorable response to BCG is suggested to be achieved in tumors exhibiting high CD8+ T-cell infiltration with elevated CD8/CD4 ratios, enhanced interferon-gamma response signatures, and favorable B-cell populations, as opposed to BCG-refractory tumors that demonstrate elevated Treg infiltration with FOXP3+, CTLA4+, IL10+ expression; macrophage polarization toward M2 phenotype; and PD-L1 overexpression [7].

The expression of Indoleamine 2,3-dioxygenase (IDO1), an immunosuppressive enzyme is studied as a promising predictive biomarker for BCG failure and cancer specific survival [20,21]. Through a multi-omics analysis of a discovery cohort (*n* = 73) and subsequent validation in an independent cohort of 75 HR-NMIBC patients, heightened IDO1 expression was pinpointed as a crucial determinant of BCG treatment failure [20]. Mechanistically, IDO1’s role in fostering an immunosuppressive tumor microenvironment is supported by its strong correlation with key immune checkpoints, including PD-1 (PDCD1), PD-L1 (CD274), and CTLA4 [21]. Additionally, it is suggested that IDO overexpression can promote EMT through the activation of the interleukin 6, signal transducer and activator of transcription 3 (STAT3) and PD-L1 signaling pathways [22]. 

Additionally, low levels of CD4/CD8 T-cells at the tumor base, the absence of a pre-existing Th2-polarized immune response in the tumor microenvironment, ARID1a mutation, and Ezrin protein over-expression have been identified as factors that correlate with BCG treatment failure [23].

A summary of genetic, molecular, immune classifications for NMIBC is presented in Table 2.

## 5. Critical Pathological Factors in BCG-Unresponsive NMIBC

### 5.1. Lymphovascular Invasion

An independent prognostic factor that significantly affects NMIBC outcomes is lymphovascular invasion (LVI), which was found in 25.7% of cases from a cohort of 245 high-grade BCG-treated NMIBC patients. Compared to LVI-negative tumors, there was a 2.28-fold increased risk of high-grade recurrence and a 2.85-fold increased risk of progression to MIBC [24].

### 5.2. Concurrent Carcinoma In Situ

Carcinoma in situ (CIS) is a high-risk variant of non-muscle-invasive bladder cancer for which BCG immunotherapy is the established first-line treatment. Evidence demonstrates that BCG with a maintenance schedule is highly effective, achieving a complete response rate of up to 84% and reducing the risk of progression to muscle-invasive disease by 35% [25].

Critically, however, the presence of CIS is itself a significant negative prognostic factor, with major clinical trial groups like the EORTC [26] and CUETO [27] identifying it as a key predictor of both recurrence and progression and it remains an argument for early radical cystectomy due to superior oncologic outcomes [28].

### 5.3. Histological Variants

Due to their different biological and immune profiles, non-urothelial histological variants often respond poorly to BCG therapy and result in worse survival outcomes [22]. When LVI is combined with concurrent histological variants (micropapillary, sarcomatoid, plasmacytoid, neuroendocrine differentiation), the hazard ratio for progression increased to 4.15, with a 5-year recurrence-free survival of only 28.6% and progression-free survival of 45.2% [24]. Consequently, aggressive surgical options like radical cystectomy are frequently recommended for these high-risk patients [28].

### 5.4. Lamina Propria Invasion

Although underreported by pathologists, the extent of lamina propria (LP) invasion in T1 NMIBC is an important determinant of BCG response [29]. As a prediction tool, extensive or multifocal (E/M) LP invasion is independently associated with a significantly higher risk of progression (HR 5.37; 95% CI: 2.2–13.1; *p* < 0.001) and correlates with poorer progression-free and cancer-specific survival [29]. In order to address this diagnostic gap, Yanagisawa et al. demonstrated that en bloc resection achieved a 100% diagnostic rate for muscularis mucosae invasion (pT1a/b), significantly outperforming the 77.6% rate of conventional methods (*p* = 0.01) and resulting in an invasion depth of ≥2 mm, making it an independent prognostic factor for progression [30].

## 6. The Current Role of Radical Cystectomy for NMIBC

Addressing the common concern that radical cystectomy (RC) severely diminishes quality of life when compared to bladder-sparing therapy (BST), the CISTO trial demonstrated the non-inferiority of 12-month physical functioning for the RC cohort (ATE, 0.9; 95% CI, −0.6 to 2.4; *p* = 0.22), with patients typically recovering from a 3-month post-operative decline to reach functional equivalence by 6 months [8]. While BST predictably offered better urinary and sexual health-related quality of life, RC was associated with superior emotional, cognitive, financial, and global health outcomes, challenging the assumption that major surgery necessarily imposes a greater economic or psychological burden than conservative therapy [8]. While these comparative effectiveness findings are clinically relevant, generalizability should be interpreted cautiously due to the observational nature of the study and due to the wide variety of BST options that continue to be approved.

A multicenter registry analysis of 141 patients undergoing RC for BCG-refractory high-risk NMIBC revealed significant clinical understaging, with 34.8% muscle invasion (≥pT2) and 16.3% lymph node positivity on the RC specimen, confirming that conventional imaging and endoscopy systematically underestimate disease extent [31]. The upstaging phenomenon after RC remains consistent, illustrating a “stage-migration” effect where early cystectomy identifies and treats occult advanced disease that can be overlooked in patients on BST [8].

Despite upstaging, only 2.1% of patients progressed to metastatic disease in the RC group, yielding 12-month and a 5-year cancer-specific survival (CSS) rates of 95.9% and 90.5% respectively [31]. With manageable perioperative morbidity (9.9% major complications; 0.7% mortality), these data support timely RC as a potentially curative therapy in appropriately selected patients that captures occult invasive disease before clinical manifestation [31].

FDA-approved BSTs like nadofaragene firadenovec (53.4–79% CR), N-803 plus BCG (71% CR), Gemcitabine-Docetaxel (50% CR), and pembrolizumab (40.2% CR) provide effective alternatives to cystectomy for BCG-unresponsive patients [32,33,34,35]. Conservative treatment options will be further expanded through combination strategies, such as immunomodulators paired with checkpoint or FGFR inhibitors or even BCG to enhance treatment durability and avoid surgical intervention [36]. However, the marked 12-month recurrence-free survival advantage with early cystectomy (96% vs. 67% with BST) in the CISTO data suggests that bladder-sparing approaches will possibly play a role dependent on disease biology and patient selection, with cystectomy retaining a vital role in appropriately selected patients [8].

## 7. Precision Framework for Cystectomy Timing in BCG-Unresponsive NMIBC

### 7.1. Patient-Centered Decision Factors

The timing of cystectomy in NMIBC or the response prediction of BCG could be esti-mated through computational integration of the presented data (Table 3), with factors such as: genomic and molecular subtyping (BRS class, UROMOL21 class, immune phenotype), clinicopathologic factors (T1 depth, concurrent CIS, lymphovascular invasion, histologic variants) and disease characteristics (papillary-only versus CIS-predominant).

Patient health status can be integrated using standardized physical functioning scores (EORTC QLQ-C30), alongside comorbidity and frailty measures (e.g., Charlson Comorbidity Index and Geriatric 8 scores) and can be used to stratify perioperative risk and competing mortality. Patient preferences can be captured through shared deci-sion-making and patient-reported outcome instruments related to bladder symptoms (ICIQ-OAB), enabling explicit trade-off discussions between recurrence risk and bladder preservation.

### 7.2. Integrated Decision Algorithm

**Figure 1:**
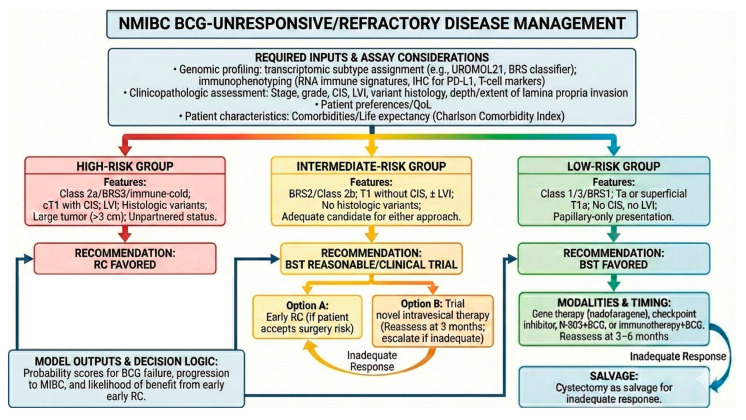
Conceptual multi-omics decision support algorithm for BCG-unresponsive NMIBC integrating molecular subtypes, immune contexture, pathological risk factors, and patient-related variables to generate probabilistic risk tiers guiding bladder-sparing therapy versus early radical cystectomy.

Putting together the advancements in bladder cancer biomarkers into a clinically relevant model, we provide a computational decision support framework for integrating biomarkers in the management of BCG-unresponsive/refractory NMIBC (Figure 1). This framework is intended as a decision support prototype rather than a validated clinical tool.

### 7.3. Required Inputs and Assay Considerations

The proposed framework assumes the availability of (i) a transcriptomic subtype assignment (e.g., UROMOL21 and/or BRS classifier) from RNA-seq or a clinically deployable targeted expression panel; (ii) immunophenotyping (RNA immune signatures and/or IHC for PD-L1 and representative T-cell markers); (iii) clinicopathologic variables (stage, grade, CIS, LVI, variant histology, depth/extent of lamina propria invasion); and (iv) patient characteristics (comorbidities and life expectancy—Charlson Comorbidity Index). For assay harmonization, minimum tumor content thresholds and batch correction/normalization procedures should be pre-specified to ensure that subtype calls are stable across platforms and centers.

### 7.4. Model Outputs and Decision Logic

The framework should generate probability scores for BCG failure, progression to MIBC, and the likelihood of benefit from early RC. Outputs can be mapped to recommendation tiers (e.g., “RC favored”, “BST reasonable/clinical trial”, “BST favored”).

### 7.5. Discordant Signals and Missing Data

Discordance (e.g., “immune-inflamed” phenotype with high-risk transcriptomic class) should be handled with explicit rules such as weighted scoring, uncertainty flags, or a “multidisciplinary review” category. Missing inputs (e.g., absent RNA data) should trigger fallbacks to established clinicopathologic-only risk models.

### 7.6. Validation Roadmap

This framework is conceptual and is not presented as a validated clinical tool. A practical next step is retrospective development in multi-institutional NMIBC cohorts with standardized endpoints, followed by external validation with calibration and decision curve analysis. Prospective evaluations should assess clinical utility (e.g., net benefit, impact on timing of RC, and patient-reported outcomes).

## 8. From Code to Clinic: Challenges and Future Directions in AI for Bladder Cancer Risk Stratification

### 8.1. Public Datasets

Public transcriptomic resources such as TCGA-BLCA (n = 412), GEO da-tasets (GSE13507, GSE32894), and the UROMOL2021 consortium (n = 834 NMIBC) provide essential platforms for transcriptomics-based classifiers needed in the development and validation of genomic and molecular biomarkers [37]. Recent integrative analyses com-bining TCGA and GEO cohorts identified 396 differentially expressed genes that differen-tiate NMIBC from MIBC, enabling progression prediction computations with machine learning and achieving AUC = 0.89 [37,38]. The 11-gene immune-related prognostic sig-nature derived from TCGA was externally validated across GSE13507 (n = 165, C-index = 0.72) and GSE32894 (n = 224, C-index = 0.69), outperforming EORTC risk tables [38,39]. Because whole-transcriptome subtyping is difficult to implement routinely, immuno-histochemistry is being explored as a practical surrogate approach for molecular subtyp-ing in clinical settings [39]. Future NMIBC precision frameworks will leverage federated learning across public datasets in order to validate composite signatures integrating mul-tiple genetic, molecular, pathologic and patient-related factors [37].

### 8.2. Challenges in AI and Computational Genomics for Bladder Cancer

While AI shows promise, a substantial gap still exists between its potential and clinical adoption [40]. Current risk stratification models for NMIBC often fail to meet clinical evidence standards, exhibiting poor discrimination for recurrence and only marginal improvement for progression [41]. Current barriers in the implementation of complex models like artificial neural networks, are their “black box” nature which hinders patient counselling, and the lack of large-scale multicenter datasets that can lead to model overfitting, preventing external validation [40]. Furthermore, models trained on older data face “decaying relevance” as clinical practice continues to evolve [41]. Methodological weaknesses is also an issue that becomes apparent when simpler regression models like LASSO often outperform complex algorithms, suggesting a premature focus on model complexity over foundational data quality [40]. This limitation highlights the need for clinically interpretable, genomics-anchored integrative models over purely algorithmic approaches.

### 8.3. Strategic Future Directions

Due to the prognostic heterogeneity of NMIBC, there is a need for more accurate risk models to overcome the failures of existing tools [41]. It is believed that computationally derived multimodal deep learning, which integrates clinicopathological features, molecular biomarkers, gene signatures, and histopathology images will enhance predictive accuracy [40,41].

To ensure clinical integration, researchers must establish rigorous standards, including the curation of large, standardized datasets and the execution of prospective clinical trials [40]. Additionally, emerging technologies like large language models may eventually support clinical reasoning and decision-making in order to transform AI and computational genomics into a validated, personalized decision-support system that improves patient outcomes [40].

## 9. Conclusions: Toward Precision Timing of Radical Cystectomy

During the last few years, we have witnessed numerous advances in NMIBC computational molecular classification and prospective comparative effectiveness evidence [37]. Genomic and molecular subtyping systems—including BRS1-3 classification, UROMOL21 stratification, and immune contexture frameworks—provide possible molecular determinants of BCG responsiveness, complementary to traditional TNM staging. Being of major clinical importance, BCG responsiveness can be predicted with the aid of tumor biology and immune phenotype with BRS3 and immune-cold tumors associated with BCG resistance.

Recent prospective data from the CISTO cohort challenges conventional assumptions regarding cystectomy: 12-month physical functioning is non-inferior between the RC and BST cohorts, with RC demonstrating potential improvement in emotional well-being and financial outcomes in the selected populations [8]. The marked recurrence-free survival advantage (96% vs. 67% at 12 months) suggests its oncologic benefit, while retrospective outcome data demonstrate that early intervention yields 90.5% 5-year cancer-specific survival [8,31]. In another cohort, outcomes were substantially worse among patients progressing to clinical MIBC prior to RC (5-year CSS 70.4%) [42].

We emphasize how bioinformatics-driven stratification models integrating a multi-omics framework of transcriptomics, genomic alterations, and immune signatures might support clinical decision-making. We proposed a computational decisional framework for the timing of cystectomy in BCG-unresponsive NMIBC that integrates molecular subtyping identifying high-risk tumors unlikely to respond to intensive intravesical therapy, clinicopathological factors predicting aggressive behavior, patient wellbeing and preferences balancing recurrence-free survival against bladder preservation, and comparative effectiveness evidence demonstrating oncologic and quality-of-life outcomes. We propose that this integrated precision framework may help stratify patients toward early radical cystectomy, identify those most likely to benefit from emerging bladder-sparing strategies, and prioritize appropriate candidates for clinical trial enrollment.

## Figures and Tables

**Table 1:** UROMOL21 molecular class characteristics and impact on clinical management [14].

Class	Molecular Characterization	Phenotype	Potential Impact on Clinical Management
1	High activity of early cell cycle genes and GATA3 regulation; frequent FGFR3 and RAS mutations;	Low EORTC risk score; exhibits the best recurrence-free survival	May benefit from intravesical chemotherapy and potentially from FGFR inhibitors
2a	High chromosomal instability; high expression of late cell cycle and DNA replication genes; APOBEC mutational signatures; TP53 pathway alterations (RB1 loss, PPARG/E2F3 gain); high FOXM1 and ESR2 activity.	Frequently T1 and high-grade tumors; associated with the worst progression-free survival	Suggestive of high-risk behavior; may warrant early cystectomy consideration in carefully selected patients; potential benefit from immunotherapy/checkpoint inhibitors due to high mutational load and neoantigen burden.
2b	High expression of EMT genes and cancer stem cell markers; high RNA-based immune score (CTLs and T helper cells); high PD\-L1 expression; ESR1, FGFR1, and STAT3 activity.	Inflamed phenotype; lower risk of progression compared to class 2a; high immune infiltration correlates with lower recurrence rates.	High PD\-L1 positivity and immune expression could suggest responsiveness to immunotherapy; may exhibit poor response to BCG.
3	High expression of FGFR3-coexpressed genes and basal markers (KRT5, CK5/6); GATA3 positive; high AR and GATA3 activity; lower gene promoter methylation and depleted immune contexture.	Associated with lower tumor stage and grade; unique phenotype characterized by androgen receptor activity.	Could be candidates for trials with FGFR inhibitors due to high FGFR3 signaling/mutations; intravesical chemotherapy could be considered as alternative.

**Table 2:** Summary of NMIBC genetic/molecular/immune classifications and risk frameworks.

Framework	Input	Intended Use	Limitation(s)
Hedegaard NMIBC expression classes [13]	Transcriptomics	Early NMIBC grouping for recurrence/progression biology	Older cohorts/platforms; limited linkage to BCG-response endpoints
UROMOL/UROMOL21 [14]	Transcriptomics (RNA-seq or deployable targeted panel)	NMIBC-specific prognostic stratification providing risk enrichment for progression	Requires assay standardization, tumor-content/batch control; prospective clinical utility and external validation still needed
BRS (BCG Response Subtypes 1–3) [7]	Transcriptomics classifier	Predict likelihood of BCG response/failure; might support RC vs further BST	Dependent on standardized classifier and harmonized platforms; needs prospective utility testing
Immune contexture phenotypes (inflamed/excluded/desert; immune signatures) [18]	RNA immune signatures ± IHC (T-cell markers/PD-L1, etc.)	Identify immune “hot/cold” tumors; complement BCG-response prediction; guide immunotherapy rationale	Immune state is dynamic and sampling-dependent; signature cutoffs vary; single-marker IHC often insufficient; limited prospective NMIBC decision validation
Genomic alteration–based groupings (e.g., FGFR3-driven vs. genomically unstable patterns) [11]	Targeted sequencing (mutations ± CNAs)	Prognosis and pathway-based stratification; identify actionable targets	Genomics alone may not predict BCG response; heterogeneous definitions; may miss expression/immune state and microenvironment effects

**Table 3:** Treatment decision matrix: early RC vs. BST.

Feature	Favor Early Radical Cystectomy (RC)	Favor Bladder-Sparing Approach
Molecular Profile	Class 2a (UROMOL21), BRS3 phenotype, immunologically cold characteristics	Class 1/3 (UROMOL21), BRS1/BRS2 phenotype, luminal phenotype, immunologically hot
Pathologic Features	T1 disease, E/M lamina propria invasion lymphovascular invasion (LVI), histologic variants	Ta or superficial T1a disease; absence of LVI or variants
Presence of CIS	Concurrent CIS (40–50% intrinsic progression rate)	Papillary-only disease
Response to Therapy	High risk of BCG failure or poor responsiveness	Potential candidates for novel BST
Patient Age/Health	Younger patients with >10–15-year life expectancy (to minimize long-term risk)	Elderly patients with multiple comorbidities or limited lifespan
Patient Preference	Priority is oncologic outcomes and prevention of upstaging	Strong preference for organ preservation; acceptance of surveillance burden

## Data Availability

No new data were created or analyzed in this study. Data sharing is not applicable to this article. All analyses were based on previously published datasets and clinical trial results available in the public domain and referenced throughout the text.

## Abbreviations

The following abbreviations are used in this manuscript:

NMIBC	Non-muscle-invasive bladder cancer
MIBC	Muscle-invasive bladder cancer
BCG	Bacillus Calmette–Guérin
BRS (BRS1–3)	BCG Response Subtypes
CISTO	Comparison of Intravesical Therapy and Surgery as Treatment Options
TURBT	Transurethral resection of the bladder tumor
EORTC	European Organization for Research and Treatment of Cancer
CUETO	Spanish Urological Club for Oncological Treatment
CIS	Carcinoma in situ
LVI	Lymphovascular invasion
RFS	Recurrence-free survival
PFS	Progression-free survival
GI	Genome instability (subtype)
AA-like	Aristolochic acid-associated (subtype)
EMT	Epithelial-to-mesenchymal transition
Tregs	Regulatory T cells
MDSCs	Myeloid-derived suppressor cells
IDO1	Indoleamine 2,3-dioxygenase
lncRNAs	Long non-coding RNAs
RC	Radical cystectomy
BST	Bladder-sparing therapy
